# An enzyme-free technique enables the isolation of a large number of adipose-derived stem cells at the bedside

**DOI:** 10.1038/s41598-023-34915-0

**Published:** 2023-05-17

**Authors:** Seher Yaylacı, Demet Kaçaroğlu, Özgür Hürkal, Alper Murat Ulaşlı

**Affiliations:** 1 Department of Medical Biology, Faculty of Medicine, Lokman Hekim University, Ankara, 06800 Turkey; 2 Plastic Reconstructive and Aesthetic Surgery, Lokman Hekim Hospital, Ankara, 06800 Turkey; 3 Physical Therapy and Rehabilitation, Faculty of Health Sciences, Lokman Hekim University, Ankara, 06800 Turkey; 4 Romatem Ankara Physical Therapy and Rehabilitation Center, Ankara, 06700 Turkey

**Keywords:** Stem cells, Regeneration

## Abstract

Adipose tissue derived stromal cells (ADSCs) play a crucial role in research and applications of regenerative medicine because they can be rapidly isolated in high quantities. Nonetheless, their purity, pluripotency, differentiation capacity, and stem cell marker expression might vary greatly depending on technique and tools used for extraction and harvesting. There are two methods described in the literature for isolating regenerative cells from adipose tissue. The first technique is enzymatic digestion, which utilizes many enzymes to remove stem cells from the tissue they reside in. The second method involves separating the concentrated adipose tissue using non-enzymatic, mechanical separation methods. ADSCs are isolated from the stromal-vascular fraction (SVF) of processed lipoaspirate, which is the lipoaspirate's aqueous portion. The purpose of this work was to evaluate a unique device ‘microlyzer’ for generating SVF from adipose tissue using a mechanical technique that required minimal intervention. The Microlyzer was examined using tissue samples from ten different patients. The cells that were retrieved were characterized in terms of their cell survival, phenotype, proliferation capacity, and differentiation potential. The number of progenitor cells extracted only from the microlyzed tissue was in comparable amount to the number of progenitor cells acquired by the gold standard enzymatic approach. The cells that were collected from each group exhibit similar levels of viability as well as proliferation rates. In addition, the differentiation potentials of the cells derived from the microlyzed tissue were investigated, and it was discovered that cells isolated through microlyzer entered the differentiation pathways more quickly and displayed a greater level of marker gene expression than cells isolated by enzymatic methods. These findings suggest that microlyzer, particularly in regeneration investigations, will allow quick and high rate cell separation at the bedside.

## Introduction

Regenerative cell therapy, or the use of stem cells to treat damaged or injured tissue, became a popular treatment option for last two decades. Because of their potential to supply a renewable reservoir of progenitor cells that may generate different cell types, tissue architectures, and even organs, stem cells are critical for tissue regeneration^1,2^. Adipose-derived stem/stromal cells (ADSCs), which were first identified in 2001 by Zuk and colleagues, have a significant regeneration potential. The aqueous fraction obtained from the enzymatic digestion of lipoaspirate is used to isolate ADSCs. This aqueous fraction, which also contains endothelial precursor cells, endothelial cells, macrophages, smooth muscle cells, lymphocytes, pericytes, and pre-adipocytes, is known as the stromal vascular fraction (SVF)^3–5^. In recent years, SVF has gained a lot of attention as a potential source for regenerative cell therapy^4,6,7^. This is because stem cells produced from adipose tissue have various advantages over other regenerative treatments. The SVF used in daily clinical settings is autologous, thus the cells included in SVF do not pose the risk of human leukocyte antigen (HLA) mismatch as allogeneic cells do^8,9^. Adipose tissue -the source of SVF- has a higher stem cell density than bone marrow, ranging from 5 to 10%, and adipose tissue extraction is less invasive than bone marrow aspiration^10,11^. Uncultured SVF can be used instantly^12,13^. Direct application is rapid and safe since it provides high stem cell potency and can be used during the surgery since no culture or manipulation is required^14–16^. ADSCs has been demonstrated to release growth factors such as epidermal growth factor (EGF), vascular endothelial growth factor (VEGF), basic fibroblast growth factor (bFGF), and keratinocyte growth factor (KGF) in addition to their vast differentiation potential^17^. Growth factors are released at bioactive quantities and have anti-apoptotic and angiogenic properties^18^. ADSCs release several cytokine molecules in addition to growth factors and have immunomodulatory and anti-inflammatory effects by interacting with immune system cells^19^.

A standard procedure for ADSC isolation has yet to be identified within the scope of the investigations conducted thus far. The “International Federation of Adipose Therapeutic and Science (IFATS)” and the "International Cellular Therapy Association (ISCT)" collect current study data in order to provide standardized guidelines. For SVF and ADSC, IFATS and ISCT have established a "dynamic document" that summarizes current literature and replaces it with new data and findings from ongoing clinical trials. SVF cells should have a viability of > 70% and ADSCs should have a viability of > 90%, according to this declaration. Colony-forming unit (CFU-F) studies indicated that the frequency of stromal progenitors in SVF was greater than 1%. CD13, CD29, CD44, CD73, CD90 positive (> 40%) and CD34 positive (> 20%) are the most common main markers found in stromal cells within the SVF. Furthermore, these cells should be able to differentiate into adipogenic, osteogenic, and chondrogenic cells^20^.

The most frequent laboratory experimental method for isolating SVF from lipoaspirate is to utilize collagenase to breakdown the collagen bounds in the fat portion of the lipoaspirate. This mechanism divides the content into two phases: adipocyte fraction at the top and cellular components at the bottom^21^. However, using enzymes like collagenase, trypsin, or dispase can be expensive, harmful in terms of safety due to microbial origin, and have a varied efficacy^22,23^. Human complement system has been demonstrated to be activated by collagenase preparations, which can result in a local inflammatory reaction^23^. Moreover, enzymatic approaches can also interfere with stem cell differentiation potency^23^.

In practically all techniques of obtaining SVF by enzymatic digestion, in which commercial equipment or kits are utilized in the laboratory or intraoperatively, washing 1 to 4 times with solutions such as physiological saline or lactated Ringer's solution, is required for deactivation and cleaning of the enzyme. The presence of residual enzymatic activity can be detected using spectrophotometric, fluorometric, and chemiluminescence methods; however, it is frequently not possible to perform this procedure while the operation is in progress due to time loss.

The clinical use of mesenchymal stem cells obtained from adipose tissue after ex vivo culturing and proliferation is highly regulated and requires high financial capacities. SVF is a heterogeneous cell mixture containing adipose tissue originated non-cultured stromal/stem cells For the SVF used in clinical settings, only minimal cell manipulation is allowed according to the Good Manufacturing Practice regulations of the European Parliament and Council (EC regulation no. 1394/2007). Additionally, the use of the enzymatic method to obtain SVF has not yet been approved by the Food and Drug Administration (FDA) in the United States, and long-term safety data of SVFs derived by the enzymatic method in various tissues are not yet available in the literature. On the other hand, purifying adipose tissue and harvesting stem cells immediately after surgery, processing SVF in sterile conditions is mandatory too. Therefore, protocols other than enzymatic digestion of adipose tissue that maintains GMP conditions are necessary for clinical use of ADSC-based therapeutics^24,25^. Mechanical rupture of tissue in a closed environment has previously been presented as an alternative technique to enzymatic digestion^26^. However, the procedures developed thus far necessitate higher quantities of lipoaspirate because the number of ADSCs isolated using these systems is significantly lower compared to the number that obtained using enzymatic techniques, while an external force applied within the scope of a mechanical system significantly reduces the number of viable cells. Furthermore, the cell compositions of SVFs derived using different approaches may differ.

With regards to enhance the cellular yield and the stem cell content included in the SVF obtained mechanically, new devices have been introduced. Among these systems, the microlyzer system consists of a hexagonal blade system with parallel blades. The adipose tissue is repeatedly passed through blade systems of varying diameters (2400, 1200, 600) to fragment tissue into microparticles and detach stromal cells from the adipose tissue in order to obtain microfragmented adipose tissue rich in progenitor cells and stromal vascular fraction. The microlyzer device applied in this study received ISO 13485 certification and CE marking. The device is registered and listed with the US Food and Drug Administration (FDA).

In this study, we aimed to compare the performance of the Microlyzer method with the gold standart enzymatic method in terms of cell number, immunophenotype and viability. Moreover, cultured cells were also investigated in vitro in terms of cell proliferation, survival and differentiation capacity.

## Materials and methods

### Materials

Collagenase NB 4 Standard Grade was was used for enzymatic digestion (Nordmark Arzneimittel Gmbh & Co. Kg, Uetersen, Germany Cat No: S1745402). ‘Next’ syringes (10 mL), 2400-1200-600-micron microlyzers were obtained from T-Biotechnology (Bursa, Turkey). Acridine Orange/Propidium Iodide Stain was obtained from Logos Biosystems, South Korea. All cell culture chemicals were purchased from Biological Industries (Kibbutz Beit HaEmek, Israel) and cell culture consumables were purchased from Nest Scientific USA Inc. MTT (3-(4,5-Dimethylthiazol-2yl)-2,5-diphenyltetrazolium bromide) was purchased from (Biotium, California, USA Cat No: 10059).

### Human adipose tissue collection and preparation

All procedures performed in this study were in accordance with the ethical standards of the institutional and/or national research committee and with the 1964 Helsinki Declaration and its later amendments or comparable ethical standards and the study was approved by the ethical committee of Medical Sciences of Lokman Hekim University Turkey (study number; 2020/048). Human lipoaspirate wastes acquired after an abdominal liposuction procedure for elective (cosmetic, aesthetic, etc.) objectives at Lokman Hekim Akay Hospital's Plastic and Aesthetic Surgery Clinic were used in this investigation. Study participants and/or their legal guardian(s) had signed a written consent form and agreed to participate in the study prior to surgery. Healthy volunteers without chronic endocrinological disease. The lipoaspiration has been performed using aspiration syringe with locking fins and single use cannula. Post-surgical lipoaspirate wastes (100 mL) were collected from ten patients (ın the power analysis, the sample size was calculated by selecting the effect size = 0.24, alpha = 0.05, and the power of the test as 90%.) (6 female/4 male) aged between 30 and 55 years (median age, 46 years) and a mean body mass index of 29 (range: 25–35). The adipose tissue was kept at + 4 °C and treated within 24 h after receiving the lipoaspirate. Lipoaspirates were separated from the tumescence solution by standing them upright for 10 min. The sample was then transferred to 10 mL Next syringes (T-Biotechnology, Bursa, Turkey) and centrifuged for 8 min at 1500×*g* in S-106 swing rotor centrifuge (T-Biotechnology, Bursa, Turkey). After centrifugation the oil layer at the top and the tumescent solution at the bottom were discarded. A total of 30 mL residual condensed adipose tissue were isolated. For each protocol 10 mL of adipose portion was divided among the various sterile injectors.

### Stromal vascular fraction isolation protocols

#### Protocol 1 (P1)

Protocol 1 was designed to compare the enzymatic and non-enzymatic microlyzer methods with solely centrifugation. In this protocol after decantation and centrifugation the pellet accumulated at the bottom of the NEXT syringe was collected. Unfortunately, the pellet had very small numbers of viable nucleated cells and we could not culture these cells in order to obtain plastic adherent stem cells. Consequently, the results of P1 protocol were not further analyzed in this study.

#### Protocol 2 (P2) enzymatic protocol

**Figure 1:**
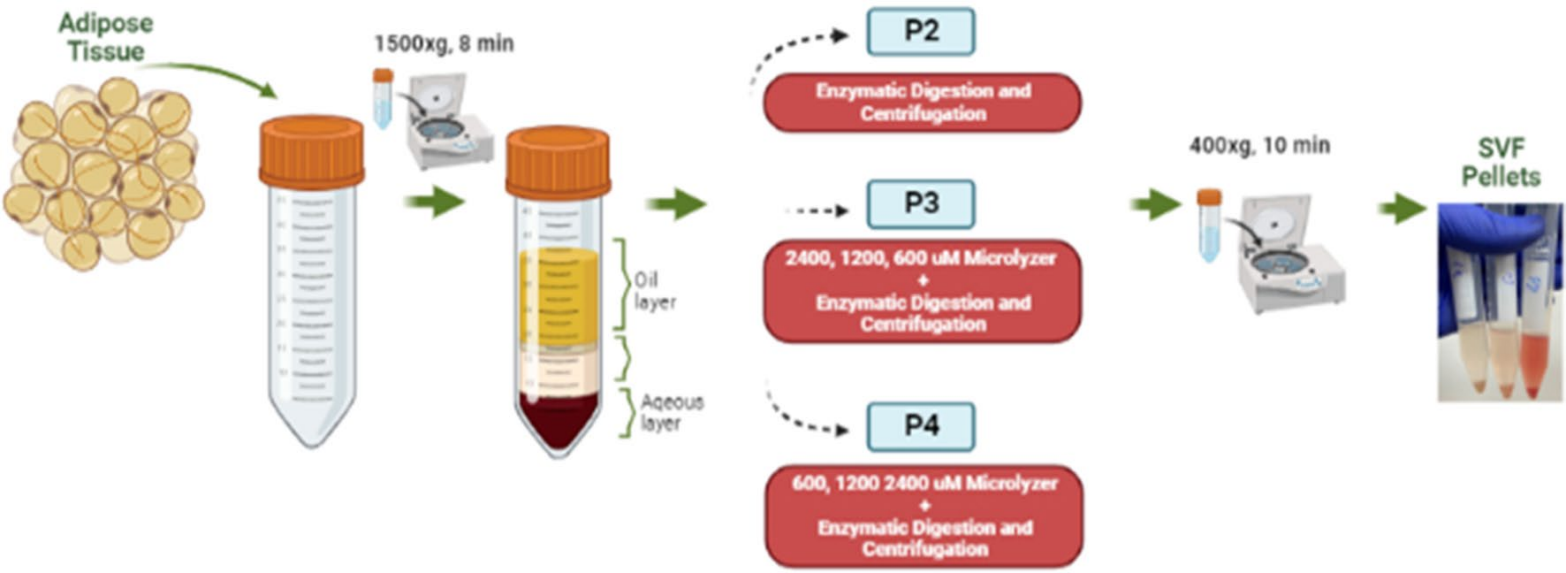
depicts the various stages involved in generating the stromal-vascular fraction (SVF) from adipose tissue.

The condensed adipose tissue (10 mL) was incubated with 0.2 U/mL Collagenase NB 4 (Nordmark Arzneimittel Gmbh & Co. Kg, Uetersen, Germany) for 30 min at 37 °C in a shaking incubator. Then it was transferred to a conical tube and centrifuged at 400×*g* for 10 min. After centrifugation, the pellet that was accumulated at the bottom of the tube was transferred to another tube. Saline was added for washing and centrifuged again. After washing step, SVF pellet was aspirated manually (Fig. 1).

#### Protocol 3 (P3, enzymatic digestion of microlyzed fat product)

The condensed adipose tissue (10 mL) was processed seven times via the 2400, 1200 and, 600-micron microlyzers (T-Biotechnology, Bursa, Turkey). Microlyzed tissue was incubated with 0.2 U/mL Collagenase NB 4 (Nordmark Arzneimittel Gmbh & Co. Kg, Uetersen, Germany) at 37 °C in a shaking incubator for 30 min. After incubation, it was transferred to a conical tube and centrifuged at 400×*g* for 10 min. Then the resulting pellet (SVF), was washed with the same amount of saline and centrifuged again at 400×*g* for 10 min. After washing step, SVF pellet was aspirated manually (Fig. 1).

#### Protocol 4 (P4-the microlyzer method)

The condensed adipose tissue (10 mL) was processed thirty-one times via the 2400 and 1200-micron microlyzer and a hundred and one times via a 600-micron microlyzer. Then, the resulting microlyzed tissue was washed with the saline and centrifuged again for 400×*g* 10 min. After washing step, the resulting pellet at the bottom of the tube was aspirated manually (Fig. 1).

Following enzymatic digestion and microlyzer disruption, the cell suspension obtained was passed through a 100 µm cell strainer in order to eliminate bulky tissue fragments and debris. This technique enables the extraction of individual cells and tiny clusters of cells that are still embedded in their original matrix.

### Hematoxylin and eosin staining

The microlyzed tissues were embedded directly in a mounting medium (Scigen O.C.T. Compound Cryostat Embedding Medium, Fisher Scientific) and snap frozen in liquid nitrogen. Sections ranging in thickness from 7 to 12 µm were sectioned using a cryostat (LEICA CM 3050S) and mounted to Super Frost R Plus slides (Thermo Fisher Scientific, USA) before being air dried for up to 90 min at room temperature. The samples were then stained for 3 min at room temperature with Mayer’s Haematoxylin (Sigma Aldrich Chemical, USA). Counterstaining was performed by staining with eosin (Sigma Aldrich Chemical, USA) for 30 s, and dehydration was completed by serial washing with 95% ethanol, 100% ethanol, and Histo-Clear (National Diagnostics, USA). Finally, slides were cover slipped.

### Cell counting and viability assessment

Stromal vascular fractions belonging to ten different donors prepared using with three different protocols was diluted 1:10 and mixed with Acridine Orange/Propidium Iodide Stain dye F23001 (Logos Biosystems, South Korea). To obtain the number of viable cells,10 µL of sample were loaded into the Luna Automated Fluorescent Cell Counter (Luna-Stem, Logos Biosystems, South Korea). The total number of nucleated and viable cells were recorded. The measurements were performed in triplicates and the mean was calculated accordingly.

### Immunophenotyping of free cells

Free cells obtained through three different protocols were analyzed in terms of cluster of differentiation (CD) protein expressions to reveal the ratio of progenitor cells. SVF’s were centrifuged at 2270×*g* for 5 min, and the supernatant was discarded and cell count was performed by Trypan Blue (Bioind, Israel). For each sample; 10^5^/mL cells were used and stained with the tagged antibodies according to manufacturer’s instruction of Human Mesenchymal Stem Cell Marker Verification Kit protocol (R&D Systems, Cat No: Fmc020). Briefly, cell samples were washed with 2 mL of staining buffer. Then for each sample 10 μL of positive antibody (antiCD90 (APC conjugated), anti CD105 (PerCP conjugated), antiCD73 (FITC conjugated)), 10 μL of the Negative Marker Cocktail (antiCD45, antiCD34, antiCD11b, anti HLA-DR and, antiCD79) (PE conjugated) and 10 μL isotype control antibody were added and incubated for 30–45 min at room temperature in the dark. Following the incubation, to remove any excess antibody cells were washed with 2 mL of staining buffer and centrifuged. The final cell pellet was resuspended in 200–400 μL of staining buffer for flow cytometric analysis. By gating on intact cells based on dot plots of forward scatter versus side scatter, cell debris was eliminated. Cell quest Pro software was used to depict fluorescence analyses as histograms (BD Biosciences, USA). Based on comparisons with isotype matched controls, the percentages of cells expressing CD90, CD73, and CD105 were calculated for each sample.

### Adipose derived stem cell culture

Stromal vascular fractions obtained with three different protocols were centrifuged at 4000 rpm for 5 min at room temperature. After removing of the supernatant, low glucose DMEM (Bioind, Israel), supplemented with 1% antibiotic/antimycotic (Biowest, France), 20% FBS (Bioind, Israel) media was added onto cells and transferred into the T-25 cell flask. After 24 h of incubation at standard cell culture conditions (37 °C, 5% CO_2_), adherent cells were washed with PBS (Biowest, France) at 37 °C and maintained in complete medium until reaching of 80–90% confluence. After 72 h, images of adhered cells were taken under an inverted light microscope (Leica Dm Il, Leica Biosystems). Adipose-derived stem cells grown in culture were used for viability, proliferationy and differentiation potential assays.

### Cell viability analysis

To determine the proliferation capacity of cultured ADSCs obtained from SVF of each protocol, 2 × 10^3^cells/well at passage number 3 were seeded onto 96-well plates and incubated at 37 °C with 5% CO_2_ for 24 h. At the end of 24 h, 48 h and 72 h, MTT reagent at a final concentration of 0.5 mg/mL (Biotium, USA, Cat No: 10059) was added onto each well and incubated at 37 °C for 4 h in the dark. After incubation,100 µL of solubilization solution was added to each well to dissolve the formazan crystals. Then absorbance was recorded at 570 nm on a microplate reader (Biotek Synergy H1, Biotek Instruments, USA) device. The measurements were performed in triplicates and the mean was calculated accordingly.

### Proliferation assay

To measure proliferation capacity, ADSCs were seeded in triplicate in six-well culture dishes (4 × 10^3^cells/well) and incubated in maintenance medium at 37 °C, 5% CO_2_ for 14 days. The culture medium was then discarded and the cells were washed three times with 1× PBS (Gibco). Cells were fixed in methanol:acetic acid for 5 min and then methanol was removed.

To stain fixed ADSCs, 0.5% (w/v) crystal violet solution was added for 15 min and then washed with 1× PBS and distilled water. The stained cells were dried at room temperature and absorbance at 595 nm was recorded on a microplate reader (Biotek Synergy H1, Biotek Instruments, Usa). The measurements were performed in triplicates and the mean was calculated accordingly.

### In vitro differentiation potential analysis

Differentiation potential of adhered ADSCs were analyzed by histological staining, immunofluorescence staining and gene expression analysis.

#### Differentiation of ADSCs into chondrogenic, adipogenic and osteogenic lineages

For adipogenic lineage analysis; ADSCs at a ratio of 6 × 10^3^ cells/cm^2^ (passage number 3) were seeded on 24 well microplate well for histological and immunofluorescence staining and on 6 well plate for gene expression analysis. To induce adipogenic differentiation, MesenCult Basal Medium and 10× and 500× adipogenic differentiation supplements medium (Stemcell Technologies, Canada) were used. The medium was replaced every 2–3 days. The number of cells were determined according to manufacturer’s recommendation.

For osteogenic lineage analysis; ADSCs at a ratio of 3 × 10^4^ cells/cm^2^ (passage number 3) were seeded on 24 well microplate well for histological and immunofluorescence staining and on 6 well plate for gene expression analysis. To induce osteogenic differentiation, MSCgo osteogenic differentiation medium (Bioind, Israel) was used. The medium was replaced every 2–3 days. The number of cells were determined according to manufacturer’s recommendation.

For chondrogenic lineage analysis; ADSCs at a ratio of 3 × 10^4^ cells/cm^2^ (passage number 3) were seeded on 24 well microplate well for histological and immunofluorescence staining and on 6 well plate for gene expression analysis. To induce chondrogenic differentiation, MSCgo chondrogenic differentiation medium (Bioind, Israel) supplemented with MSCgo chondrogenic differentiation supplementary mix (Bioind, Israel) (1:10) was used. The medium was replaced every 2–3 days. The number of cells were determined according to manufacturer’s recommendation.

#### Histological staining

After 14 or 21 days of culture, cells were fixed in 4% PFA for 30 min at 4 °C and then blocked with 1% BSA solution for one hour. Adipogenic differentiation samples was stained with Oil red-O solution (SİGMA ALDRİCH CHEMİCAL CO, USA) for 5 min. Then cells were washed with 1× cold PBS three times. Osteogenic differentiation samples were stained with Alizarin red solution (Sigma Aldrich Chemical CO, USA) for 40 min in the dark. Then cells were washed with distilled water three times. To demonstrate chondrogenic differentiation, samples were stained with Safranin O solution (Sigma Aldrich Chemical CO, USA) 5 min. Then cells were washed with 1× cold PBS three times. All samples were imaged using inverted light microscope (Leica Dm Il, Leica Biosystems).

#### Immunofluorescence staining

For immunofluorescence staining, ADSCs were seeded on glass slides (Ø 13 mm) at a previously mentioned ratio. After 14 days of culturing, the differentiation medium was removed and cells were fixed in 4% PFA (Serva, Germany) for 10 min. After fixation, cells were washed with 1× PBS three times. Then for permeabilization, 0.1% Triton X-100 (Serva, Germany) was added and kept for 10 min and then cells were washed with 1× PBS three times. To reduce non-specific binding, cells were blocked with 1% bovine serum albumin (BSA)/PBS. To detect expressed protein of interest for specific lineage commitment, cells were incubated at overnight at 4 °C with anti-aggrecan antibody (Abcam, USA Cat No: Ab36861) at a 1:100 dilution in 0.1% BSA/PBS, anti-PPAR gamma antibody (Abcam, USA Cat No: Ab36861) at a 1:200 dilution in 0.1% BSA/PBS and anti-osteopontin antibody (Abcam, USA Cat No: Ab8448) at a 1:200 dilution in 0.1% BSA/PBS. Then to detect primary antibodies goat anti-rabbit IgG H&L Alexa Fluor 488 (Abcam, USA, Cat No: Ab150077) was used as a secondary antibody at a ratio 1:200 in 0.1% BSA/PBS. Then cover glass were mounted with Fluoromount W (Serva, Germany). Then cells were imaged under up right fluorescence light microscope Olympus BX43 (Olympus, UK).

#### Gene expression analysis

Total RNA was isolated using TriZOL (Invitrogen, USA). RNA recovery and purity were evaluated with the Nanodrop 2000 system (Thermo Fisher Scientific, USA). cDNA was synthesized and amplified (BIORAD, USA) using the OneStep qRT-PCR Kit (Invitrogen, USA). According to the manufacturer's instructions, the reaction was set as; 55 °C for 5 min, 95 °C for 5 min, and 95 °C for 40 times 15 s, 60 °C for 30 s, and 40 °C for 1 min. For each reaction, amplification of the glyceraldehyde-3-phosphate dehydrogenase (GAPDH) transcript was be included as an internal control. qPCR melt curves was generated and analyzed after qRT-PCR to assess product specificity for each gene. The marker transcripts listed in Table 1 was used to determine specific gene expression profiles. The comparative Ct method with efficiency correction was used to analyze the results^27^. Accordingly, expression rates higher than one indicate up-regulation of the gene of interest, while ratios less than 1 correspond to down-regulation. The analysis was repeated at least 3 times for each experimental group.

**Table 1:** RNA primers used in gene expression analysis.

Gene name	5′→3′ oligo sequence
RUNX2	F: GTCCATCCACTCTACCACCC R: TGAAATGCTTGGGAACTGCC
COL1A1	F: GCTACTACCGGGCTGATGAT R: ACCAGTCTCCATGTTGCAGA
AGGRECAN	F: TACACTGGCGAGCACTGTAA R: GTACTTGTTCCAGCCCTCCT
COL2A1	F: CCAGCAAACGTTCCCAAGAA R: GCGTAGGAAGGTCATCTGGA
ADIPONECTİN	F: CCTGGTGAGAAGGGTGAGAA R: ATGGGCATGTTGGGGATAGT
FABP4	F: GGGCCAGGAATTTGACGAAG R: AAGTGACGCCTTTCATGACG
GAPDH	F: GTCTCCTCTGACTTCAACAGCG R: ACCACCCTGTTGCTGTAGCCAA

### Statistical/data analysis

The mean and standard error (SEM) was calculated for all variables. Normality test was used to determine whether the distribution of all experimental group variables was consistent with a Gaussian distribution. Differences between cell groups was tested with the non-parametric Wilcoxon Matched-Pairs Signed Ranks test. In all analyses, the difference was considered statistically significant if the associated p value is less than 0.05. Calculations was made with GraphPad Prism (Version 9; Graphpad Software, San Diego, Ca, USA).

## Results

### Total number of free cells and cellular viability ısolated by different protocols

**Figure 2:**
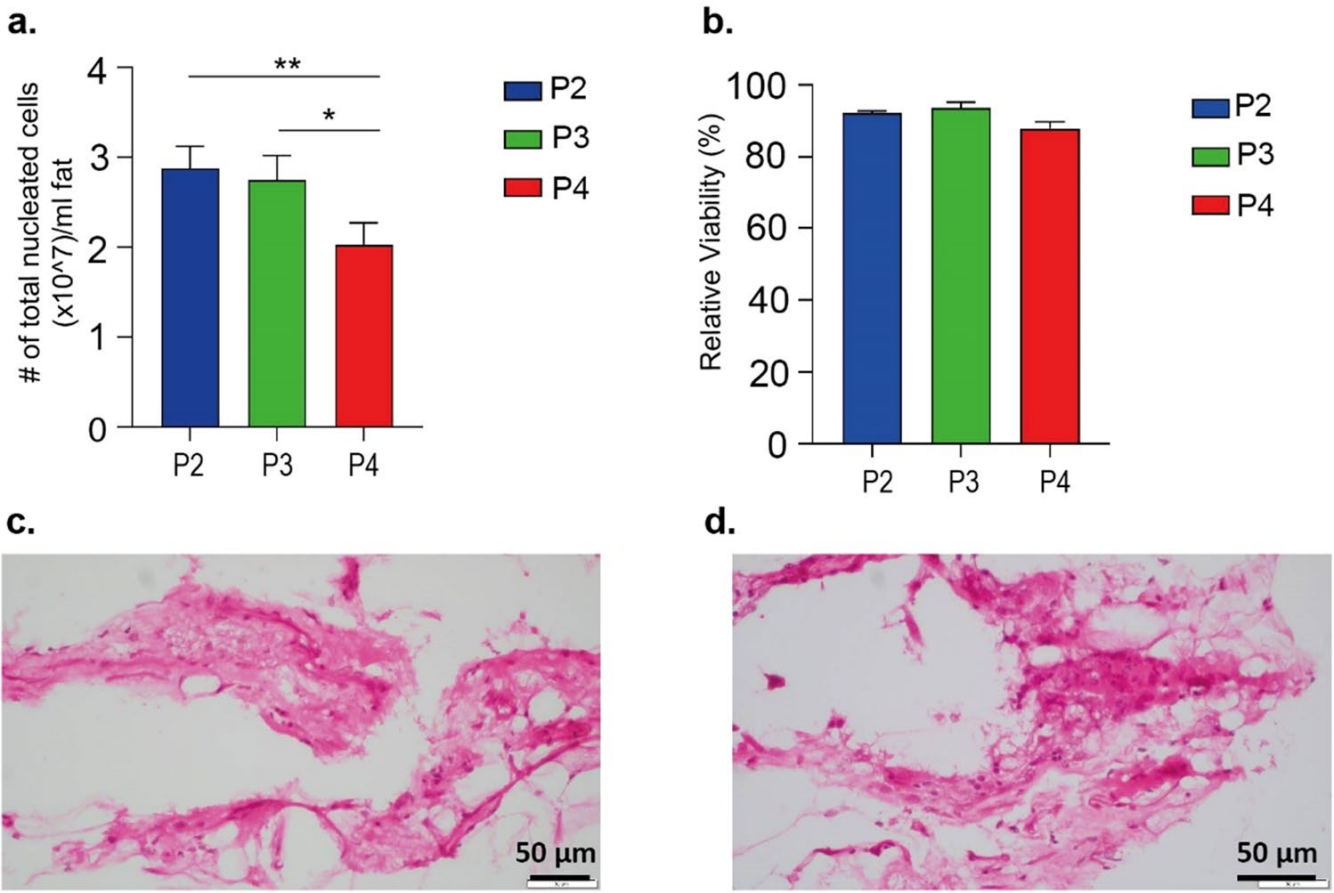
Proportion of free nucleated ADSCSs (**a**) and relative cell viability of free ADSCSs (**b**) retrieved according to different protocols. Morphological structure of stromal vascular faction isolated by protocol 3 (**c**) and protocol 4 (**d**). Values represent mean ± SEM, n = 10 (***p < 0.0001, **p < 0.01, *p < 0.05).

Within the purpose of this study, the stromal vascular fractions isolated using the 3 different procedures described in the methodology part were examined for cell quantity and viability before being cultured. The method of using the collagenase enzyme is considered as the gold standard and was employed as a control in this study. In the other procedures, progenitor cells were isolated from adipose tissue using a specially developed microlyzer. Isolations were initiated with 5 mL of condensed fat tissue for P2 and 10 mL for P3 and P4, and cell counts and percent viability were calculated from the SVFs collected at the end of each protocol. In Protocol P3, after microlyzer process, 16% of condensed tissue was trapped in the blades and this percentage was 21% for P4. Consequently, a mean of 8.4 mL of nanofat was digested enzymatically in P3 and a mean of 7.9 mL of final fat product was obtained after microlyzer processing and centrifuged in P4. The calculation of nucleated cells obtained from 1 mL of condensed fat were performed using initial amount of fat (10 mL). The enzyme-using methods (P2 and P3) yielded approx. 2.8 × 10^7^ cells per 1 mL of condensed fat, while the enzyme-free method yielded approx. 2 × 10^7^ cells per 1 mL of condensed fat (Fig. 2a). Furthermore, the viability of the cells obtained was approximately approx. 90%for each technique (Fig. 2b). Additionally, the SVF isolated with P3 and P4 underwent histological analysis to observe tissue and cell organization after being processed by microlyzer. According to histology images taken after isolation, it is observed that there were both free cells and bounded cells that were residing in their niche which was a dense connective tissue (Fig. 2c,d).

### Characterization of surface markers of free isolated cells

**Figure 3:**
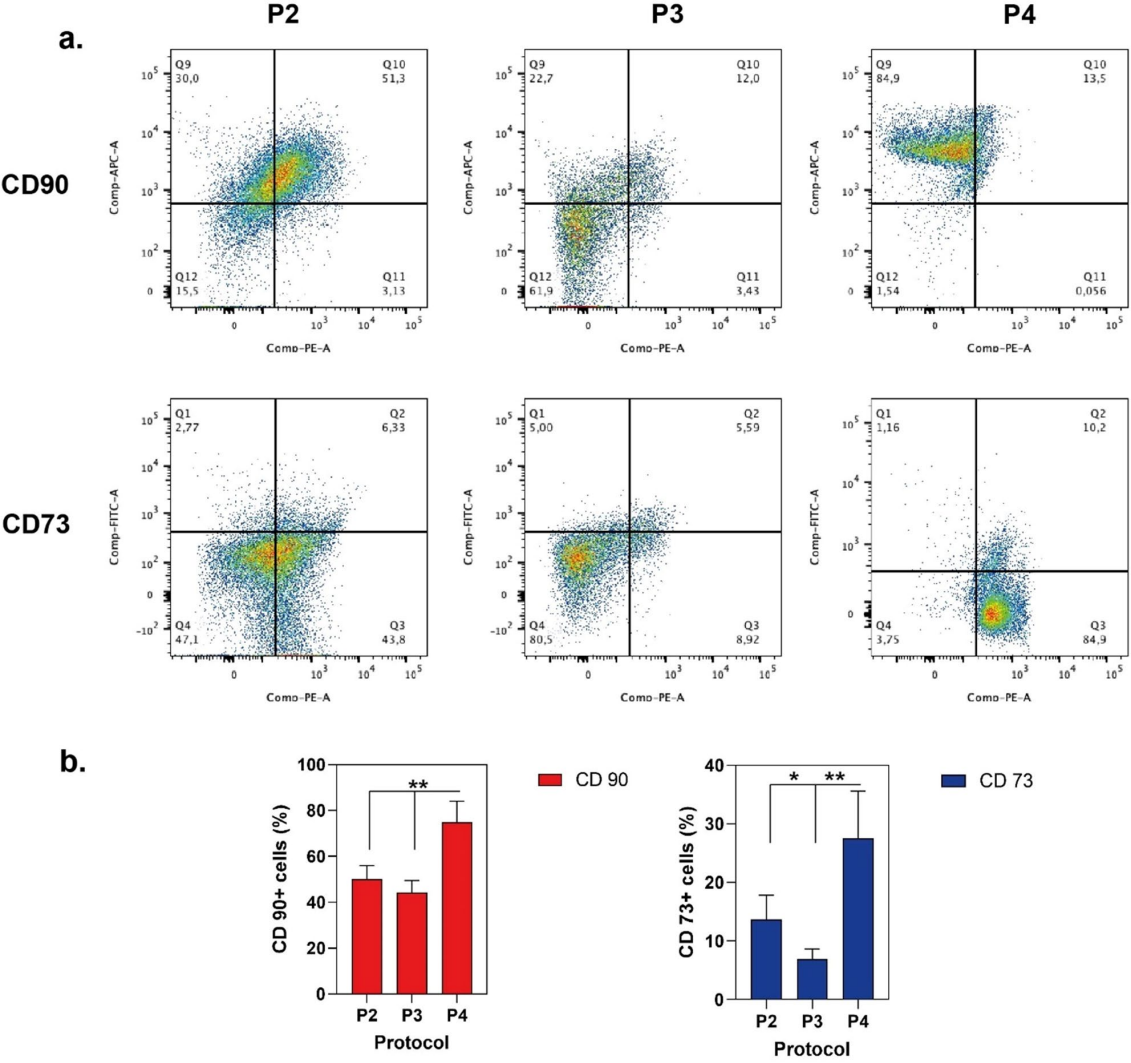
Expression of surface markers detected by flow cytometric analysis of free cells from SVF. (**a**) Data show a representative set of dot-plot from one individual in each isolation protocol. (**b**) The percentage of positive cells for CD90 and CD73 marker was calculated after subtraction of the non-specific fluorescence obtained with the control (unmarked). Values represent mean ± SEM, n = 10 (***p < 0.0001, **p < 0.01, *p < 0.05).

For the cell surface immunophenotyping, free cells collected through each protocol were examined using the “Human Mesenchymal Stem Cell Marker Verification Multi-Color Flow Cytometry Kit”. Based on positive (CD90, CD105, CD73) and negative (CD45, CD34, CD11, CD79, and HLA DR) markers, cells were identified, stained, and characterized. P4 cells had the statistically significant higher percentage of CD73 and CD90 expression than other cell types isolated by other protocols P2: CD73+ 13.71%, CD105+ 0.1%, CD90+ 45.97%, P3: CD73+ 6.06%, CD105+ 0.1%, CD90+ 40.41%, P4: CD73+ 27.51%, CD105+ 0.5%, CD90+ 69.7%, (Fig. 3a,b).

### Stemness characterization of isolated cells

**Figure 4:**
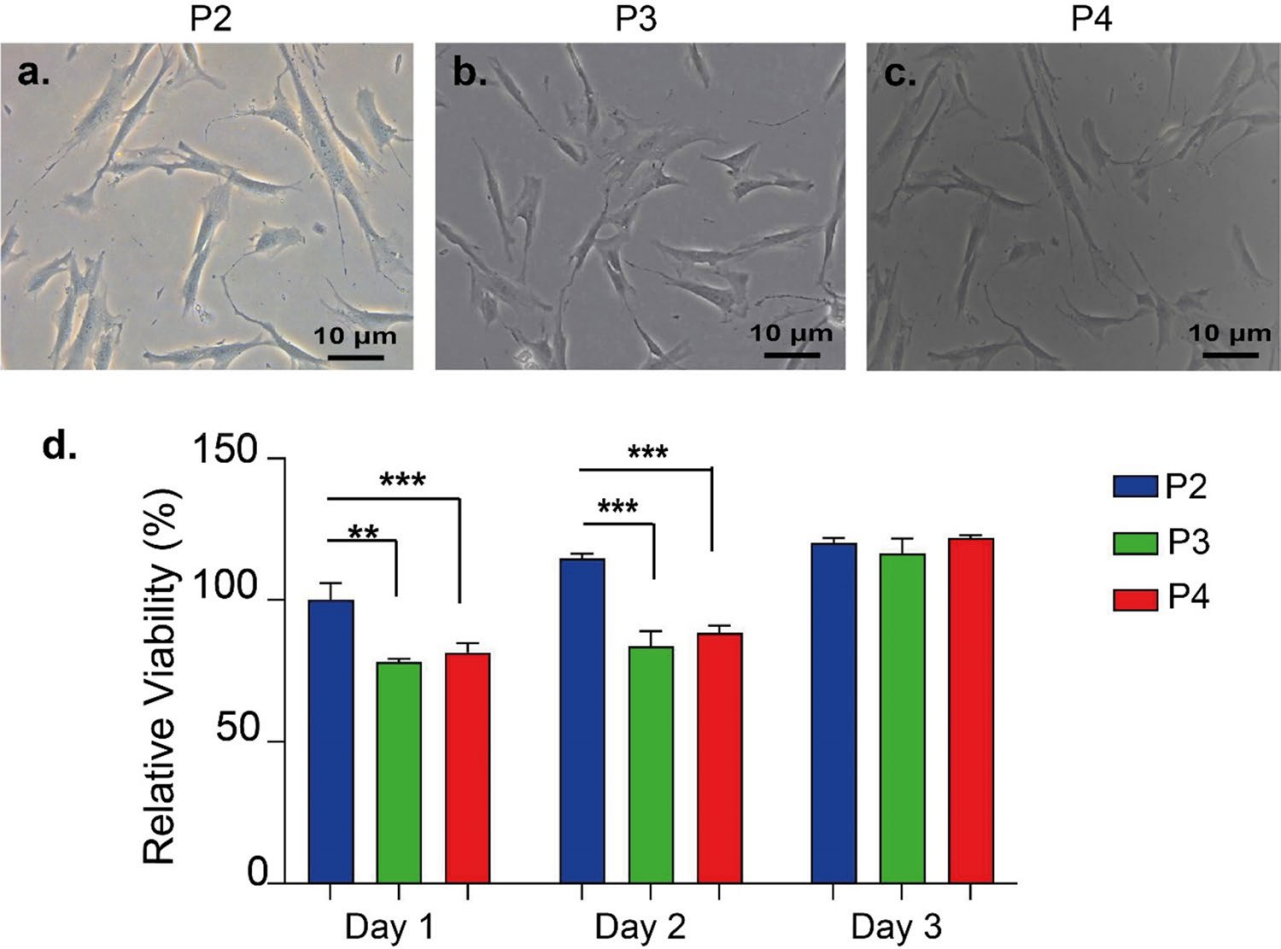
Light Microscope images of ADSCSs isolated from SVF with the use of protocol 2 (**a**), 3 (**b**) and 4 (**c**) at passage 0 (**d**). Viability analysis of ADSCSs isolated by protocol 2, 3 and 4 on day 1, 2 and 3. Values represent mean ± SEM, n = 10 (***p < 0.0001, **p < 0.01, *p < 0.05).

**Figure 5:**
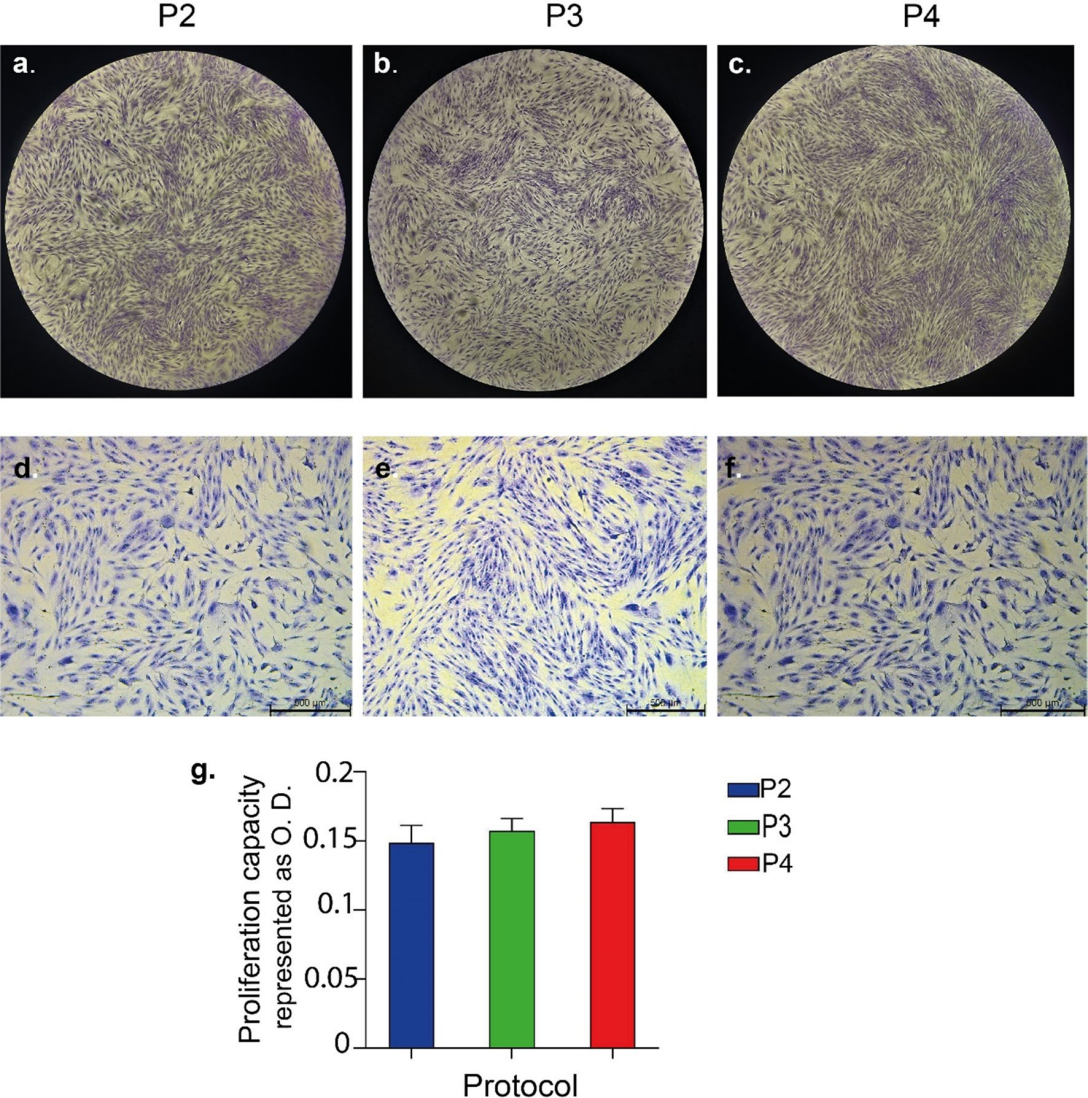
Image and analysis of cultured isolated ADSCS. Representative phase-contrast microscopy images and crystal violet staining photographs from 4x (**a**–**c**) and × 10 objectives (**d**–**f**) of stained ADSCSs on day 14 at passage number 3. Bar graph showing proliferation capacity of ADSCSs according to OD values. The data is represented as mean ± SD (n = 3; *p < 0.05) (**g**).

To evaluate the stemness potential of isolated cells, cells were cultured on cell culture plastics (Fig. 4a–c). The viability of cells on tissue culture plastic was assessed and all group of isolated cells showed similar viability results (Fig. 4d). Before proceeding on to the trilineage differentiation experiments, the proliferation capacity of the cells was also assessed. Cells reaching passage number 3 were transferred to well plates to be cultured for 14 days. At the end of the 14th day, there was no quantitative and morphological significant differences between the cells isolated using the enzyme and the cells isolated with the microlyzer in terms of the proliferation rate (Fig. 5).

**Figure 6:**
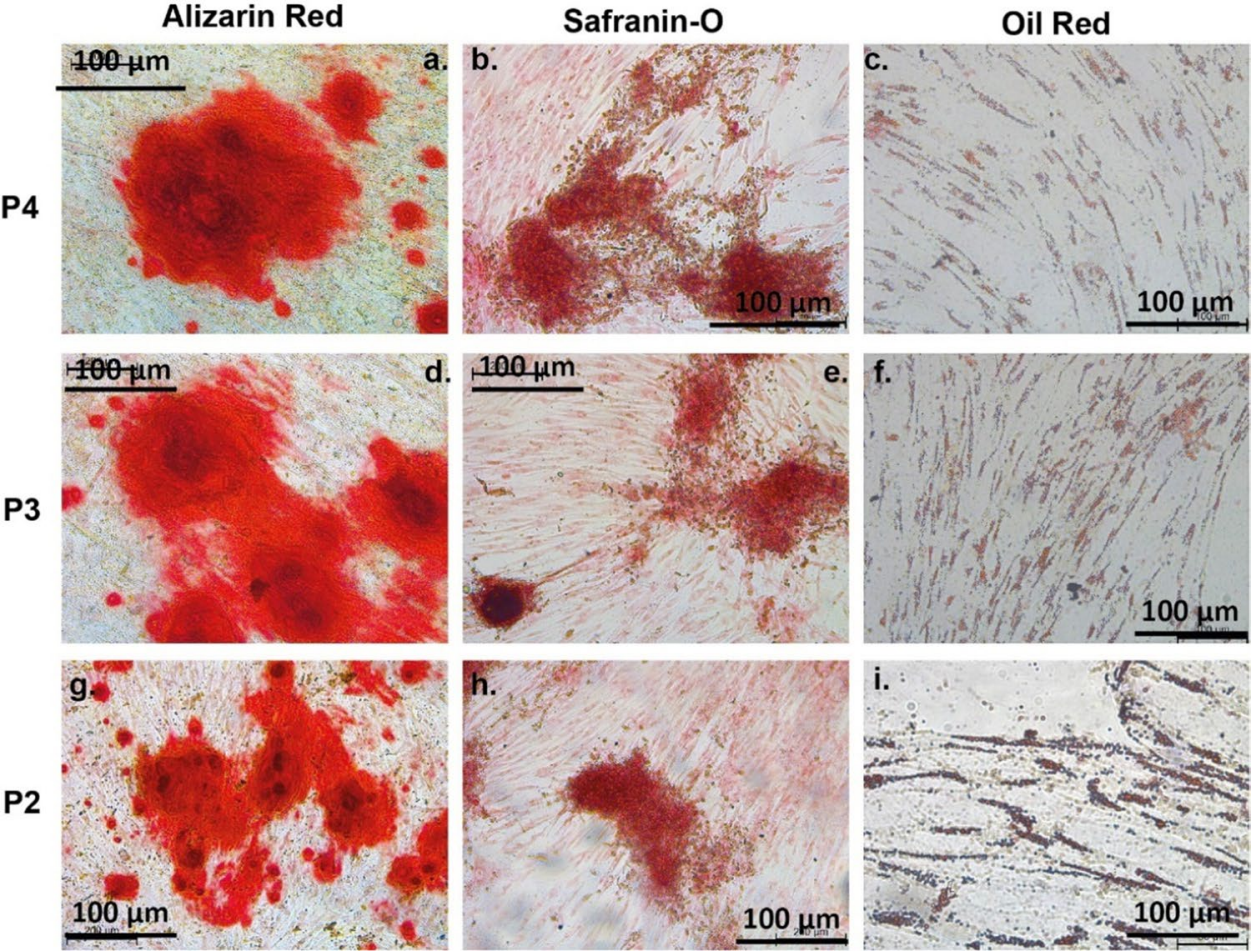
Trilineage differentiation of passage number 4 of ADSCS isolated by different protocol. Differentiation of human ADSCSs isolated with P2: (**a**–**c**), P3: (**d**–**f**) and P4: (**g**–**i**) to adipocyte, chondrocyte and osteoblast lineages. Adipogenesis was indicated by the accumulation of lipid vacuoles stained with Oil Red O; (**c**,**f**,**i**) chondrogenesis was indicated by the deposition of sulfated proteoglycan-rich matrix stained with Safranin O (**b**,**e**,**h**); osteogenesis was indicated by extracellular matrix calcification stained with Alizarin red S (**a**,**d**,**g**). ADSCSs cultured in adipogenic, chondrogenic and osteogenic media for 13 days.

**Figure 7:**
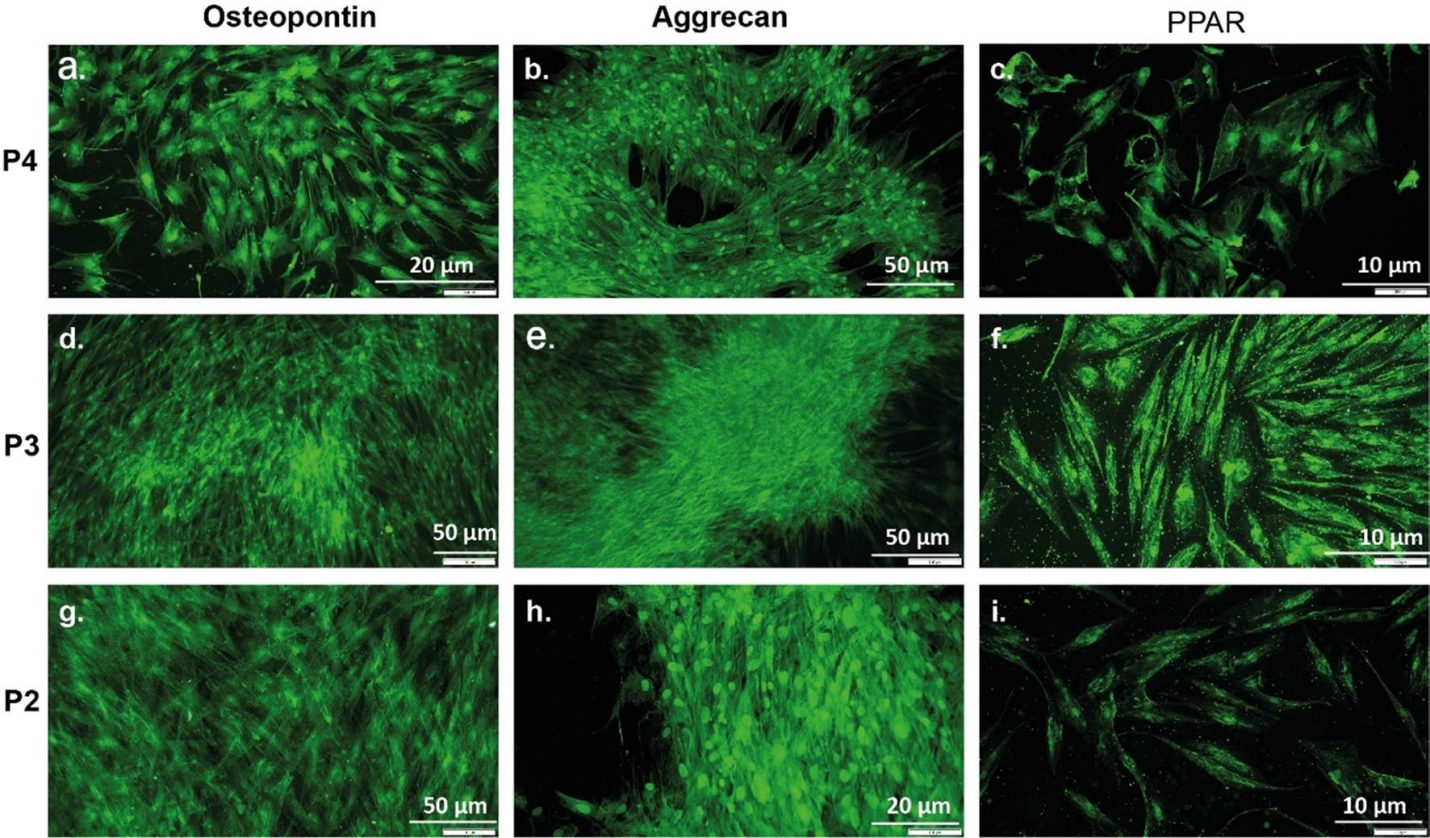
Immunofluorescence staining of human ADSCSs isolated by different protocols specific proteins three groups (**a**–**i**). Osteopontin (**a**,**d**,**g**): Aggrecan (**b**,**e**,**h**) and PPAR gamma (**c**,**f**,**i**) were labeled with Goat Anti-Rabbit IgG H&L (Alexa Fluor 488) (green).

All P2, P3, and P4 cells grown in the appropriate differentiation conditions for 14 days appeared to dye positively for each dye showing the successful commitment of progenitor cells to specific lineages (Figs. 6 and 7).

Osteopontin was used to identify cells in osteogenic medium, while Aggrecan was used to identify cells in chondrogenic medium, and antibodies specific for PPARγ were used to mark cells in adipogenic medium. As shown in Fig. 6, cells isolated using various differentiation protocols exhibited positive staining for Osteopontin, Aggrecan, and PPARγ, respectively.

**Figure 8:**
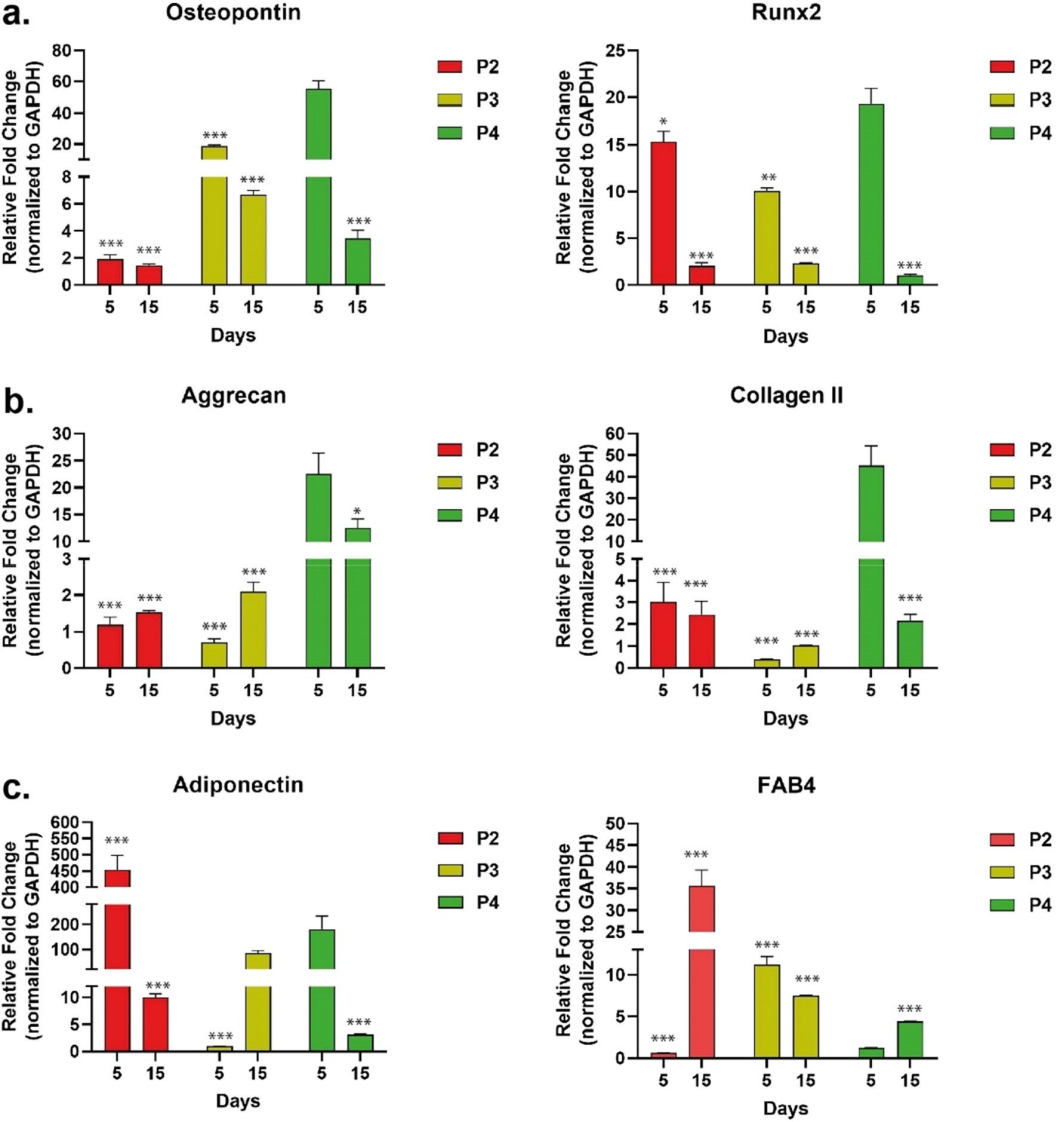
Gene expression analysis as a trilineage differentiation of ADSCSs isolated by different protocols for day 5 and 15. (**a**) Osteopontin and Runx2 genes are used for osteogenic differentiation markers, (**b**) Aggrecan, Collagen II genes are used for chondrogenic differentiation markers and (**c**) Adiponectin and FAB4 are used for adipogenic differentiation markers. The expression level of each gene was normalized against control undifferentiated samples and GAPDH was used as the internal control. Values represent mean ± SEM, n = 3 (***p < 0.0001, **p < 0.01, *p < 0.05, statistical differences between P4 D5 and other groups had shown only on figure).

Gene expression analysis were conducted for following markers; Aggrecan and Collagen II for chondrogenic commitment, Osteopontin and Runx2 for osteogenic commitment and Adiponectin and FAB4 for adipogenic commitment. According to the results, in the time-dependent expression analysis of bone markers, osteogenic differentiation was induced on the 5th day in all groups and the rate decreased on the 15th day. When the groups were compared among themselves, P4 showed a higher expression rate on the 5th and 15th days compared to P2 and P3 (Fig. 8a). On day 15, cartilage differentiation occurred in all groups, considering both marker expression rates. However, on the 5th day, the P4 group showed a statistically significantly higher expression rate than the other groups in terms of both markers while Aggrecan expression was not upregulated on the 5th day in both P2 and P3 groups (Fig. 8b). When the adipogenic markers Adiponectin and FAB4 were examined, the P4 group was again upregulated on both the 5th and 15th days. While the Adiponectin expressions of the P2 and P3 groups increased from the 5th to the 15th day, the pattern was reversed in P4, it was higher on the 5th day and lower on the 15th day. FAB4 expression was upregulated in all groups on the 15th day (Fig. 8c).

## Discussion

Microlyzer is a sterile, closed system that permits high-throughput isolation of progenitor cells at the bedside without the use of enzymes. Using a parallel filter/blade system, Microlyzer isolates cells from a small sample of adipose tissue by mechanical disintegration. The current study demonstrated that microlyzer method reveals comparable number of viable nucleated cells with enzymatic method, however the progenitor cell percentage included in the SVF prepared with microlyzer was higher. *Furthermore, the stemness and differentiation potential of the stem cells isolated with microlyzer method were equal or superior to the manual enzymatic method.*

In this study, cellular characterizations of the SVFs obtained with microlyzer approach were conducted and were compared with the gold standard enzymatic approach group. First, the number of nucleated and viable cells were analyzed. The number of nucleated cells obtained with the microlyzer system was approximately 2 × 10^7^/mL cells per 1 mL of condensed adipose tissue. According to studies published in the literature, the number of isolated nucleated cells ranges from 5 × 10^5^ to 2 × 10^6^ per 1 mL of adipose tissue, and only 2–10% of total cells has been defined as ADSCs^28,29^. The cell yields of SVF obtained with P3 in which the microlyzed adipose tissue was treated with collagenase and the enzymatic method (P2) revealed similar nucleated cell counts. Even, a mean 1.6 mL of 10 mL adipose graft was trapped inside the microlyzer, -in addition to its mechanical harvesting effect- the microlyzer process increased the surface of the fat graft and allowed the interaction between the collagenase and the graft that leads more efficient digestion of the collagen bounds in the ECM. Considering P4, in which we did not use collagenase and the adipose graft was centrifuged after passing through 2400-, 1200-, and 600-micron pored microlyzers, the nucleated cell yield was approximately 75% of the enzymatic method. The lower cell yield might partly be explained due to trapped adipose graft during microlyzer processing (a mean of 2.1 mL of 10 mL condensed fat). In the study by Zuk et al., they used washed lipoaspirate and after the liquid portion is discarded they called it processed lipoaspirate^30^. Most of studies performing manual enzymatic digestion for SVF production followed this method and used the processed lipoaspirate portion. In our study we centrifuged the lipoaspirate using higher centrifuge speed and force (1500 G) in order to discard the liquid portion of the lipoaspirate. After condensation of the adipose lipoaspirate, we obtained oil layer at the top and tumescent liquid at the bottom. We further processed the adipose layer in between these two layers which was equal to approx. 30% to 50% portion of initial lipoaspirate volume. We obtained much higher number of nucleated cells in both enzymatic digestion and microlyzer methods compared to previous reports^28^. This could be partly explained with that we used condensed adipose tissue instead of lipoaspirate. We also kept the lipoaspirate in upright position (decantation) for ten minutes in order to discard tumescent solution, however, we could not find the decantation duration in the study by Zuk et al.^30^. Furthermore, the tumescent solution included in the lipoaspirate might differ among adipose grafts depending on the amount of tumescent solution used, the type of cannula, the time period between the application of tumescent solution and the beginning of lipoaspiration.

The percentage of the viable cells obtained using the enzymatic and microlyzer methods which were assessed immediately after isolation, were similar. In this case, it can be concluded that the mechanical stress applied with the microlyzer method does not adversely affect the cells and does not reduce their viability. As can be seen from the histology results, there are both free cells and cells embedded in the ECM in the microlyzed tissue obtained. Therefore, cells partially maintain their cellular interaction with the ECM and this interaction stabilizes them.

We surmise that, microlyzed adipose tissue have a higher therapeutic potential for regeneration than enzymatically extracted ADSCs, since ADSCs in their own niche extend the cellular long-term survival. With regards to CD90 and CD73 stem cell markers, a large proportion of the cells obtained are progenitor cells, and this ratio is statistically higher in P4 which microlyzer was used to harvest stem cells. It can be said that the microlyzer method performs an enrichment of progenitor cells. *Following these analyses, the stem cell potential of the acquired cells was evaluated by seeding them on plastic cell dishes. On plastic cell dishes, cells obtained *via* microlyzer exhibited lower cellular viability for the first two days and then it reached to the enzyme group on the third day.* In the first 48 h, the cells may have difficulties adhering to the plastics due to the dense *ECM plaque that surrounds them, resulting in a low viability rate.* In the experiments analyzing the potency of stem cells, cells from all groups proliferated and gave rise independent colonies and entered the adipogenic, chondrogenic, and osteogenic differentiation pathways. When the gene expression results were evaluated together, it is observed that the cells obtained from each of the three protocols enter three different differentiation pathways, albeit at different times. An important point to be mentioned here is that it is known that cells entering the differentiation pathway begin to synthesize different markers at different times, and this situation will have an effect on the temporal difference in the expression of markers. When the groups are compared with each other, a significant finding indicates that the cells isolated with microlyzer in group P4 commit to the osteogenic and chondrogenic pathways more swiftly. Moreover, these cells often exhibited higher expression levels.

The hypoxia-inducible factor (HIF) involved signaling pathway is activated under hypoxia^31^. The previous literature revealed that HIF protein, especially HIF-1α, has been associated with MSC migration and differentiation^32^. HIF-1α acts on MSCs via the SdF-1/cXcr4 molecular axis^33^. Although HIF-1 α is expressed as a response to hypoxia, it has been also shown that HIF-1α can be activated by mechanical stimulation. Shimomura et al. reported that HIF-1α and aggrecan expression was significantly enhanced with mechanical stimulation under hypoxia but not normoxia in their study evaluating the effect of mechanical stimulation on chondrocytes^34^. Furthermore, in the study by Feng et al., authors showed that HIF-1α can be activated in endothelial cells by mechanical low shear stress, leading to enrichment of HIF1α expression at atheroprone regions of arteries under-normoxic-conditions^35^. SDF-1/CXCR4 signaling is critical for the recruitment of MSCs to the fracture site during healing. In their study, Wei et al. reported that the mechanical stimulation produced by the pressure waves of low intensity pulsed ultrasound therapy on bone enhanced MSCs migration mediated by SDF-1/CXCR4 pathway^36^. In our study, we used microlyzers for extracting stromal cells from the adipose tissue. The adipose graft was exposed to both pressure and shear stress during pushing forward and backwards through 2400–1200 and 600 micron blades. We hypothesized that, more swift commitment to the osteogenic and chondrogenic pathways and higher chondrogenic and osteogenic gene expression of the cells isolated with microlyzer in group P4 might be due to that they were exposed to the highest pressure and shear stress. The pressure and shear stress could activate HIF 1 alfa/SDF-1/CXCR+ pathway.

Compared to conventional or automated dissociation methods of adipose tissue using enzymes, the microlyzer method is faster and more cost-effective for obtaining SVF. The shorter time required for the SVF obtainment procedure with microlyzer is necessary, as it enables the surgeon to use it during surgery, or shorten the duration of the use of operating room and minimize the costs. Moreover, the distribution of the nucleated cells particularly the portion of the progenitor cells included in the SVF will determine the effectiveness of the biologic treatment. It should be expected that the higher progenitor cell yield will be associated with increased regenerative effect of the final product at the target tissue.

Lipoaspirate contains tumescent fluid, adipose tissue fragments, blood, and SVF. *SVF is considered as a heterogenous mixture of adipose-derived stem cells, red-blood cells, leucocytes, pericytes, smooth muscle cells, fibroblasts, and vascular endothelial progenitor (3–5).* In their review, Aronowitz et al., compared various enzymatic digestion and mechanical isolation techniques for obtaining SVF^28^. The residual collagenase activity and cell counts were different among the methods reviewed. Among automated/semiautomated enzymatic methods, Tissue Genesis Cell isolation system (701,000/mL lipoaspirate, 80.7% vitality), the GID SVF platform (719,000/ mL, 83% vitality) and the Celution system (360,000/mL, 84.7% vitality) resulted in higher SVF nucleated cell counts from 1 mL of lipoaspirate^37,38^ Doi et al. reported similar nucleated cell yield between Tissue genesis system and manual methods (701,000 and 702,000 per 1 mL lipoaspirate) with similar cell vitality^39^. The mechanical isolation systems revealed lower number of nucleated cells compared to automated/semiautomated and manual SVF isolation methods as the highest yield was 240,000/mL^28^. However, recently Cohen et al. compared two new nanofat processing methods; LipocubeNano (Lipocube, Inc., London, Uk) in comparison to Tulip’s NanoTransfer (Tulip Medical, San Diego, Ca). Nanofat is not yet fully defined, but in general, is thought of as fat parcel sizes of 600 microns or less^40^. The two nanofat products were further processed with enzymatic digestion and the cell count and vitality outcomes were compared. The digested adipose tissue was centrifuged at a speed of 300×*g* and duration of 5 min, and the SVF pellets were obtained. The study did not include a gold standard manual enzymatic procedure for SVF production as control. LipocubeNano resulted in higher cell counts (2.24 × 10^6^/mL), whereas Tulip’s NanoTransfer method resulted in a lower cell count at 1.44 × 10^6^/mL. Cell viability was the same (96.05%) in both groups. These two protocols were similar with P3 protocol in the current study.

In their study, Dongen et al., the authors compared the Fractionator -a device containing 1.4 mm holes, used to fractionate the adipose tissue in order to obtain SVF- and enzymatic methods^41^. They used condensed adipose tissue which they obtained after decantation and one step centrifugation. The adipose tissue was then fractionated and centrifuged again in the study group. Enzymatic digestion was performed to the two groups. The steps were similar to the P2 and P3 protocols of our study. Enzymatic isolation of the fractionated-SVF and controls resulted in a mean number of cells of 2.7 × 10^6^ and 3.5 × 10^5^ per 1 mL of condensed fat, respectively. To compare, we obtained 20 × 10^6^ nucleated cells per 1 mL of condensed tissue with microlyzer method.

Bianchi et al. compared nonexpanded Lipogems fat product with lipoaspirate^25^. Both the adipose lipoaspirate processed using Lipogems and washed lipoaspirate were digested using collagenase. The SVF obtained using Lipogems was found to have higher percentage of CD146^+^/CD90^+^/CD34^−^ expressing pericytes, and CD90^+^/CD29^+^/CD34^−^ expressing mesenchymal cells compared to lipoaspirate. The overall percentage of the cells expressing CD90 were 43.4% and 45.2% and percentage of the cells expressing CD73 were 26% and 7.1% for Lipogems and lipoaspirate, respectively. However, the authors did not report the nucleated cell counts of each procedure, thus we could not compare the nucleated cell outcome. In our study, the percentage of CD45^−^, CD34^−^, CD11b^−^, CD79^−^, and HLA^−^DR^−^, but CD73^+^ cells were 13.71% for enzymatic protocol, 6.06% for enzymatic digestion of microlyzed fat, and 27.52% the fat processed with only microlyzer. The percentage of CD45^−^, CD34^−^, CD11b^−^, CD79^−^, and HLA^−^DR^−^, but CD90^+^ cells were 45.97% for enzymatic protocol, 40.41% for enzymatic digestion of microlyzed fat, and 69.7% the fat processed with only microlyzer. The reported percentage of CD73 and CD90 expression in the study by Bianchi et al. was covering all cells in the SVF, however, in the current study we analyzed the cells negative for expression of CD45 and CD34, consequently we could not compare the data.

In the study by Tiryaki et al., they compared enzymatic method, mechanically obtained SVF with lipocube device, and the stromal vascular matrix (SVM) in regards to nucleated cell count^40^. They defined SVM as the mixture of SVF from mechanical digestion and adipose buffy coat with high ECM content -which is the lowest part of the adipose fraction after centrifugation. The SVM was treated with collagenase. The nucleated cell counts were 1.52 × 10^6^/mL in manual enzymatic method, 1.14 ± 1.33 × 10^6^/mL for SVM, and 0.67 ± 1.69 × 10^6^/mL for SVF obtained mechanically using Lipocube. The nucleated cell yield of the mechanical method was approximately 45% of manual enzymatic method while this ratio was 75% for SVM. In our study, the nucleated cell counts in the SVF obtained using microlyzer method was approximately 75% of manual enzymatic method. Considering the relative percentage of the cells obtained mechanical and enzymatic methods, it seems that the microlyzer alone can reveal similar nucleated cells with SVM in which enzyme is used to further disintegrate the cells from the ECM.

In the study by Conde-Green et al. the authors compared enzymatic digestion of lipoaspirate with centrifugation and vortexing in order to obtain stromal vascular cells from lipoaspirate^42^. The enzymatic digestion revealed 2.3 × 10^5^ cells per 1 ml of lipoaspirate while the centrifugation method revealed tenfold fewer cells. Apart from this study, we used condensed adipose tissue which is obtained after centrifugation of the lipoaspirate, for cell calculation. And we performed a second centrifugation after enzymatic digestion. This might explain the higher number of nucleated cells with our study. The number of the nucleated cells in the SVF obtained with microlyzers were also much higher compared to the centrifugation protocol in the study by Conde-Green et al., as we used two steps of centrifugation and three different sizes of microlyzers between centrifugations in order to distract the cells from the adipose tissue.

## Conclusion

Due to the time and regulatory restraints, the enzymatic procedure is incompatible with clinics. There have been numerous attempts to develop a mechanical approach with a yield comparable to collagenase. Unfortunately, none of them have performed at the same level thus far. With this study, we demonstrated that the microlyzer approach can be employed instead of the enzymatic method in terms of cell yield and viability, particularly in regenerative applications, by rapidly isolating a high rate of progenitor cells.

## Data Availability

All data generated or analyzed during this study are included in this published article.
